# Dysgeusia in Patients with Breast Cancer Treated with Chemotherapy—A Narrative Review

**DOI:** 10.3390/nu15010226

**Published:** 2023-01-01

**Authors:** Marianna Pellegrini, Fabio Dario Merlo, Elena Agnello, Taira Monge, Andrea Devecchi, Valentina Casalone, Filippo Montemurro, Ezio Ghigo, Anna Sapino, Simona Bo

**Affiliations:** 1 Department of Medical Sciences, University of Torino, 10126 Torino, Italy; 2 Dietetic and Clinical Nutrition Unit, Città della Salute e della Scienza Hospital, 10126 Torino, Italy; 3 University of Gastronomic Sciences, 12042 Pollenzo, Italy; 4 Clinical Nutrition and Dietetics Unit, Candiolo Cancer Institute, FPO-IRCCS, 10060 Candiolo, Italy; 5 Breast Unit, Candiolo Cancer Institute, FPO-IRCCS, 10060 Candiolo, Italy; 6 Pathology Division, Candiolo Cancer Institute, FPO-IRCCS, 10060 Candiolo, Italy

**Keywords:** breast cancer, dysgeusia, dysosmia, taste alterations, chemotherapy

## Abstract

Breast cancer (BC) is the most common cancer worldwide. Chemotherapy (CT) is essential for the treatment of BC, but is often accompanied by several side effects, including taste alterations, due to different mechanisms. Although dysgeusia is usually underestimated by clinicians, it is considered very worrying and disturbing by cancer patients undergoing CT, because it induces changes in dietary choices and social habits, affecting their physical and psychological health, with a profound impact on their quality of life. Several strategies and therapies have been proposed to prevent or alleviate CT-induced dysgeusia. This review aimed to evaluate the available evidence on prevalence, pathophysiological mechanisms, clinical consequences, and strategies for managing dysgeusia in BC patients receiving CT. We queried the National Library of Medicine, the Cochrane Library, Excerpta Medica dataBASE, and the Cumulative Index to Nursing and Allied Health Literature database, performing a search strategy using database-specific keywords. We found that the literature on this topic is scarce, methodologically limited, and highly heterogeneous in terms of study design and criteria for patient inclusion, making it difficult to obtain definitive results and make recommendations for clinical practice.

## 1. Introduction

Breast cancer (BC) is the most common cancer occurring worldwide in 2.26 million people in 2020 [1]. Depending on the stage of BC, treatment strategies include different chemotherapeutic agents (both alone or in combination), radiation, surgery, or hormonal therapies [2]. Chemotherapy (CT) can be used before (neoadjuvant CT) or after (adjuvant CT) the surgical procedure, during the maintenance phase, or even in palliative context in patients with metastatic tumors [3]. CT is often accompanied by side effects, including fatigue, nausea, vomiting, anorexia, diarrhea, xerostomia, dysphagia, dysosmia, and dysgeusia [4]. Dysgeusia is variably defined as an abnormal or impaired sense of taste, an unpleasant alteration of taste sensation, or a distortion or perversion of the sense of taste [5]. Because a standardized method for assessing taste alterations is lacking and dysgeusia is frequently self-reported, its rates widely range between 53% and 84% in BC subjects under treatment [6], which is in line with the 45–84% prevalence reported in other cancers [7]. Dysgeusia is closely linked to changes in olfaction as both taste and smell are involved in producing the sense of flavor [4], and the most common taste disorders reported concern the “bitter” and “sour” quality of taste [8]. Several mechanisms contribute to taste alterations in cancer patients, such as cancer-related inflammation, adverse effects of chemotherapy and of other drugs used to treat complications, nutritional status, and the lifestyle habits of the patients [9]. CT is associated with dysgeusia through multiple mechanisms, such as damage to taste buds and olfactory receptors, impairment to neurotransmission, and other physical (i.e., xerostomia) or psychological effects [10]. Dysgeusia in cancer subjects undergoing CT is usually underestimated or even ignored by clinicians, as this condition does not represent a life-threatening event nor determine drug dose modifications [9,11,12]. However, it is considered one of the most worrisome and disturbing side effects [13]. The present narrative review aims to address the following topics:

- Prevalence of dysgeusia by specific BC therapy;
- Clinical consequences of dysgeusia and its impact on quality of life;
- Strategies for the management of dysgeusia.

## 2. Materials and Methods

The following databases were queried from 1st January 1990 to 30 October 2022: PubMed (National Library of Medicine), the Cochrane Library, Excerpta Medica dataBASE (EMBASE), and the Cumulative Index to Nursing and Allied Health Literature (CINAHL). The search strategy was performed using combinations of database-specific subject headings and keywords related to “breast cancer” plus terms related to “taste alterations” (i.e., dysgeusia, smell, taste, dietary changes) and “cancer therapies” (i.e., chemotherapy, drug side effects) or quality of life. No restrictions were placed during the search. Hand-searching the references of the studies and reviews of the field was performed to augment the search strategy. Few papers were available. Therefore, all the research articles were considered: systematic reviews and meta-analyses, randomized controlled trials (RCTs), human observational studies, and case studies.

## 3. Results

### 3.1. Dysgeusia during Chemotherapy

Taste and/or smell are not altered in BC patients before CT [14]. In BC subjects under CT, the prevalence and incidence of dysgeusia are both highly variable, ranging respectively from 59% to 83% [15,16,17] and from 53% to 97.3% [7,18] (Table 1). Possible reasons for these differences are described below. Although all antineoplastic drugs have been associated with some degree of taste alterations, an observational study conducted in 151 cancer patients (35% with BC) undergoing CT reported anthracyclines, paclitaxel, carboplatin, and docetaxel as the CT agents producing the highest taste disturbance rates, while the use of cisplatin and 5-fluorouracil resulted in the lowest complaints [19]. Taxane-based therapies were reported to cause more severe dysgeusia, both in all cancers and in BC [17,20], with a prevalence of 75–93% in the former and 81.5% in the latter [17]. Two multicenter, phase III, randomized trials compared adjuvant cyclophosphamide, methotrexate, fluorouracil (CMF) with docetaxel (DXL) chemotherapy in ≥65-yo BC patients [21,22]. After the first cycle of CT, both studies reported a higher incidence of dysgeusia in the docetaxel group than in the CMF group (25% DXL vs. 11.3% CMF [21] and 23% DXL vs. 6% CMF [22]). An exploratory descriptive study on 25 adult women with BC under CT with docetaxel or paclitaxel found a higher incidence of dysgeusia in the docetaxel group (73%) when compared to the paclitaxel group (27%) [23]. Estimating the real prevalence of dysgeusia is difficult due to various factors, such as the variation in the methodology (subjective or objective) used for taste and smell assessment, the presence of confounding factors (such as the use of concomitant drugs) and the type of cancer. Indeed, very few studies have addressed the relative prevalence or severity of taste alterations following CT by cancer type both for all neoplasms [24] and for BC [6]. In BC patients, the high variability of incidence and prevalence of dysgeusia is probably related to the lack of standardized methods of assessment [20], which can lead to different detection rates of dysgeusia [6]. Most studies [15,16,18,20,21,22,23,25,26,27,28] reported subjective assessments of taste alterations as self-reported by questionnaires or interview, and only few [6,8,14,17] measured the taste recognition thresholds with objective methods, such as taste strips. Taste strips are filter paper discs impregnated with different concentrations of taste solutions of the tastes “sweet” (sucrose solution), “sour” (citric acid or tartaric acid solution), “salty” (sodium chloride solution), “bitter” (quinine-hydrochloride solution), and “umami” (monosodium glutamate solution) [6,8,14,17]. Although objective methods are superior for assessing taste physiology and impairment, subjective reports more accurately describe the experiences of cancer patients [15]. Subjective findings do not always correspond to the data derived from objective measurements and often overestimate the degree of dysgeusia, because self-reported taste alterations mostly refer to the overall flavor of food (combination of taste, smell, texture, and temperature) [17]. In addition, CT toxicities that are neither life-threatening nor require changes in the planned anticancer therapy are underestimated by clinicians, as reported by chemotherapy adverse effect information extracted from medical records [11,12]. A cross-sectional study including 92 BC women under CT with docetaxel, epirubicin, and cyclophosphamide, reported that the prevalence of dysgeusia perceived by oncologists and patients was 4.5% and 87%, respectively [11]. Accordingly, an Italian prospective multicenter study in a cohort of 556 adult BC women reported an incidence of dysgeusia after the first cycle of adjuvant CT of 8% (as perceived by physicians) and 50% (as described by the patients) [12]. However, the presence of self-reported taste alterations more reliably predicts variations in the dietary behavior of patients [6]. Both smell and taste can be affected by age. Older people have fewer olfactory receptor cells, which are more susceptible to neurotoxic substances, with less regenerative potential [14]. Therefore, smell alterations are more pronounced than taste alterations with advancing age. On the other hand, older people exhibited a lower age-related chemosensory ability and their reduction in taste perception may be less evident than in the younger subjects [15]. The time of dysgeusia onset after CT is variable in BC patients. Dysgeusia has been described both early after the first cycle [15,21,22], either after 4–7 days [6,17,23] or within 2 weeks [18,25], and with a delayed onset of up to 10 weeks [15]. Dysgeusia may be persistent or intermittent [15], with cyclic impairments and improvements of taste function [15,24]. BC patients generally recover sense of taste within 3–6 months after CT [16,20,25,29,30], reaching baseline values on average at 12 months after treatment completion [20,25]. An impairment of all basic tastes (sweet, salt, bitter, sour, and umami flavors) was reported together with an abnormal metallic taste sensation [20]. The perception of salty quality has been reported as the most compromised [8,29], especially in patients treated with taxanes [29]. Intriguingly, the presence of dysgeusia has been related to concomitant adverse events from other drugs. Patients who experienced metallic taste reported constipation and appetite loss (especially for red meat and protein food), while patients with impairments in salty, sweet, and bitter flavors reported nausea and mucositis [20]. In the presence of multiple taste alterations, nausea, diarrhea, constipation, and mucositis were frequently described [20]. Current evidence is, however, limited and does not allow firm conclusions to be reached concerning the occurrence, severity, and quality of dysgeusia under different CT regimens. Further investigations are needed to define the taste alteration occurring under specific treatments to improve patient information and preventive approaches [7].

### 3.2. Pathophysiological Mechanisms

Taste is mediated by clusters of heterogeneous taste receptors cells (TRCs) organized as taste buds on the tongue, which transmit taste information from the oral cavity to the brain via the gustatory sensory neurons of the VII, IX, and X cranial nerves [31]. TRCs have a high mitotic rate, from 10 days to ~6 weeks, being continually renewed from stem cells [31]. The pathophysiological mechanisms of CT-induced dysgeusia in patients with cancer is still unclear due to the heterogeneity in the characteristics of the studied patients, the large variations in the reported symptoms (often self-reported), the lack of a standardized method or specific biological markers to detect taste alterations, and the multifactorial etiology of dysgeusia [5,7]. The link between cancer and dysgeusia may be due to conditions, such as the characteristics of the disease itself, the side effects of drugs, and the habits of the patients. Indeed, cancer is characterized by a generalized inflammatory state, often exacerbated by infections due to immunosuppression, with the release of numerous pro-inflammatory cytokines and chemokines, which may exert their actions both locally, causing taste bud dysfunction, and at brain level, modulating the areas involved in the control of feeding behavior including taste perception [9]. It has been demonstrated that the enhanced expression of cytokines in taste buds can cause taste-related disorders and increase programmed cell death of taste cells when interferon (IFN)-α and IFN-γ are activated [32]. Among the mechanisms of post-CT taste alterations, the following have been proposed: olfactory receptor cells and taste bud damage, peripheral and central neuropathy, such as neuronal cell deterioration and neurotransmission blockage (involving the VII, IX, and X cranial nerves), mucosal injury, xerostomia, concomitant infections/inflammations requiring specific therapies, and psychological effects [10,20]. Establishing the precise mechanisms through which each drug or therapeutic scheme impairs neurosensorial cells is difficult [7]. Post-CT dysgeusia seems to be due to either the reduction of the number of receptor cells or the impairment of neural transmission [5]. Indeed, cytostatic and cytotoxic chemotherapeutics interfere with the cellular metabolism of both malignant cells and normal tissues with a high mitotic index [25]; therefore, since olfactory and taste receptor cells have a short lifespan and a rapid turnover rate of ~7 and 10 days, respectively, this explains their special susceptibility to the toxic effects of CT [5,33,34]. TRCs may be damaged and even destroyed by antineoplastic agents a few days after the administration of a single CT cycle, explaining the early onset of dysgeusia after CT and the rapid recovery after therapy discontinuation [6,17,23]. Furthermore, the impairment in neurotransmission may be secondary to the damage of cranial nerves (e.g., demyelination of nerve fibers), the modification of afferent pathways due to the cytostatic crossing through the blood-brain barrier, as well as the drug neurotoxic effects, such as the chemotherapy-induced neuropathy [5,7]. The salty taste is the one affected most frequently, while bitter and metallic tastes are also frequently reported as CT side effects. This may be due to different reasons, such as the presence of metallic- or bitter-tasting components in the structure of the drugs reaching the taste receptors by diffusion through capillaries (e.g., platinum contained in cisplatin and carboplatin drugs) [5,35,36], the production of carbonyls by the lipid peroxidation of oral epithelial cells causing metallic taste sensations [5], and the reduced threshold for metals described in cancer patients undergoing CT [35]. The iron-containing compounds in red meat can exacerbate these impaired sensations, explaining why many cancer patients experience a bitter or metallic taste during its consumption and prefer the use of plastic instead of metal cutlery and utensils during meals [35].

Zinc is a micronutrient that is mostly localized in the tongue taste buds and plays a major role in taste perception [37]. Taste and smell functions are under the control of growth factors, which stimulate stem cells in both taste buds and olfactory epithelial cells. Zinc is one of these growth factors and is a component of the salivary enzyme carbonic anhydrase VI, which stimulates sensory stem cell growth and differentiation [38]. Zinc depletion is related to the change in taste perception because it induces both damage to taste cells with a delay in TRCs turnover [9,37] and carbonic anhydrase VI deficiency leading to dysgeusia [38]. A meta-analysis of 36 observational studies, including 5747 patients (2369 with BC), found a statistically significant reduction of serum zinc concentrations in BC patients than in healthy controls, supporting the above reported mechanism [39]. Accordingly, a small observational study on 23 women with gynecological cancers who underwent gustatory tests before and after CT, exhibited a progressive reduction in serum zinc levels and concomitant impairment in taste perception [37]. A lack of serum iron and copper has also been hypothesized to cause taste disorders, but very little data are at present available [37]. Saliva is an important factor playing a fundamental role in the gustatory perception. In a prospective observational study on 45 BC patients under adjuvant CT with cyclophosphamide, epirubicin, 5-fluorouracil (CEF) or cyclophosphamide, methotrexate, 5-fluorouracil (CMF), 64% of the patients reported xerostomia by temporary salivary gland hypofunction, which lasted up to 1 year after treatment [25]. Hyposalivation induces xerostomia, which may lead to oral dysesthesia and impairment of taste perception, mastication, and swallowing because it predisposes a patient to dental caries, gingivitis, mucositis (reported in approximately 40% of BC patients under CT), changes in oral microflora with increased risk for infections, such as candidiasis [25,40,41]. In particular, oral Candidiasis may induce phantogeusia (i.e., a sensation of taste that is not produced by an external stimulus) and oral burning sensations, compromising the eating habits of the patients and impacting their nutritional status [42]. In cancer patients, nicotine or alcohol abuse or insufficient oral hygiene aggravate dysgeusia [5,8]. Adverse reactions to CT, such as gastroesophageal reflux, infections, and the drugs employed to manage these side effects or other concomitant diseases, such as proton pump inhibitors (i.e., omeprazole, lansoprazole) and antibiotics (i.e., ciprofloxacin, levofloxacin, metronidazole, tetracycline, ampicillin) can, by themselves, cause taste disturbances [43].

#### 3.2.1. Cyclophosphamide

Cyclophosphamide (CYP) is an alkylating chemotherapy drug with cytotoxic properties that damages DNA and induces oxidative stress in normal and cancer cells. It is one of the first employed chemotherapy agents and is still used as part of a cocktail of drugs to treat BC [32]. For humans, CYP is generally administered in combination with other antineoplastic drugs; in animal studies, it is used alone, which offers the possibility of better exploring the mechanisms implicated in CYP-induced dysgeusia. In mice, CYP has been reported to induce taste loss with both direct and indirect effects. Taste loss has been assessed by behavioral tests focused on taste acuity (i.e., the ability of mice to discriminate between the taste qualities of two substances), on taste sensitivity (i.e., the detection thresholds for perceiving substance taste), and on taste bud morphological tests (i.e., the number and alteration of the taste buds) [44]. Indeed, CYP can directly induce the destruction of the lingual epithelium and the death of sensory cells within taste buds, raising the taste thresholds and impairing the ability to discriminate tastes [44,45,46]. CYP suppresses the normal taste cell replacement process, resulting indirectly in a later disturbance when aging gustative cells die without replacement [45]. CYP can cause the damage of other structures such as salivary glands and von Ebner glands, causing xerostomia and consequently hypogeusia [45]. A single moderate dose of CYP induced in mice an increase in the proinflammatory cytokine tumor necrosis factor alpha (TNF-α) in a subset of type II taste sensory cells between 8 and 24 h postinjection, with a slow decline thereafter [32]. Fractionated dosing of CYP (5 doses of 15 mg/kg) prolonged the suppressive effects of CYP on cell proliferation and renewal of taste sensory cells in mice taste buds with respect to a single dose (75 mg/kg) of CYP. Fractionation also reduced the total number of cells and the proportion of type II cells within the taste buds [46].

#### 3.2.2. Monoclonal Antibodies

A phase III, randomized, double-blind, placebo-controlled trial on 808 patients with HER2-positive locally recurrent, unresectable, or metastatic BC, found that the addition of pertuzumab to the trastuzumab and docetaxel CT induced more frequently dysgeusia when compared to placebo. In the intervention groups compared to placebo, dysgeusia was reported in 16.8% versus 14.8% in patients ≤65 years old and 27.9% versus 20% in patients ≥65 years old [27]. No data are available on the possible mechanisms by which these drugs induce dysgeusia, to the best of our knowledge.

### 3.3. Impact on Health and Quality of Life

Dysgeusia is a serious problem in patients with cancer because it leads to lack of appetite, food aversion, and loss of the hedonic value of food, with a negative impact both on health and social interactions. The resulting changes in daily habits and social isolation may lead to physical and psychological distress resulting in a deterioration in quality of life (QoL) [24]. Notably, taste alterations can contribute strongly to malnutrition by impairing eating behaviors, and both weight loss and weight gain have been described as consequences of dysgeusia. Weight loss may derive from a reduced intake of foods and drinks [29,47]. Indeed, among the main reasons for decreased oral food intake, dysgeusia has been reported in 42.2% of cancer patients [48], while weight gain may be due to the consumption of strongly flavored, tasty, savory, and energy-dense comfort foods as well as sweetened drinks [20,23,49]. However, a recent prospective study [50] aiming to investigate the relationship between taste alterations and changes in food habits and body weight among 182 women with early BC during adjuvant CT reported that patients changed their dietary habits mostly to follow the World Cancer Research Fund (WCRF) recommendations (i.e., maintain a healthy weight, be physically active, eat wholegrains, vegetables, fruit and beans, limit ‘fast foods’, red and processed meat, sugar-sweetened drinks, and alcohol consumption [51]. Interestingly, in the same study despite a significant reduction in the consumption of certain foods (i.e., bread, red meat, added sugar, alcohol, and others), regardless of the presence of dysgeusia, body weight remained stable in 71.4% of the patients and was not influenced by dysgeusia [50]. Malnutrition is highly prevalent in BC patients on CT, with percentages of ~20% undernourished patients [48] and ~20–30% of overnourished patients [52]. Malnutrition in both its form was reported to be an important predictor of sarcopenia and increased infection risk, morbidity, mortality, poor treatment response, and CT toxicity [20,29,53]. Patients with BC usually receive chemotherapy for several months, during which in addition to dysgeusia, they experience other adverse effects such as nausea, vomiting, anorexia, and xerostomia which can affect their eating habits and worsen their global health. Indeed, the lack of eating enjoyment leads to inadequate energy and essential nutrient intakes, resulting in malnutrition, reduced compliance with treatment regimens, impaired immunity defense, and, ultimately, poor prognosis [4]. Moreover, taste alterations may adversely impact the daily habits and social lives of cancer patients [54], with a highly negative influence on QoL and self-esteem in BC patients [49]. Several studies reported a worsening of QoL as a consequence of CT-related taste and smell alterations in BC patients [3,11,13,16,22,30,53,55,56]. The overall impact of CT-related dysgeusia on QoL is hard to be determined because items from questionnaires generally include different adverse effects, such as xerostomia, odynophagia, dysphagia, and emotional and social aspects, making it difficult to disentangle the effects specifically linked to the alteration of taste [5]. In 299 older BC women treated with adjuvant CT with docetaxel or CMF, a worse QoL and more frequent/severe dysgeusia were found in patients treated with docetaxel compared to CMF [22]. In observational studies on patients undergoing CT (both patients with BC only and BC patients plus patients with other-than BC), a direct correlation between dysgeusia and lower QoL was reported particularly during the first two cycles of CT [30,53], with a recovery at 6-months after CT in most of the patients except for patients receiving trastuzumab [16]. An association between dysgeusia and “worse pain” and “discomfort indicators” [53] as well as the worsening of the domains of “role” and “social aspect” [13,16] were also found. BC diagnosis was related to a subjective higher distress and greater impact on daily life due to taste changes when compared to other cancers [55]. Finally, the avoidance of certain foods, such as chocolate, fruit, and coffee, may concur in affecting QoL [29]. Furthermore, the physical disability of BC patients by worsening body image and future perspective can have a profound negative psychological impact [22,53], often forcing changes in their social and relational habits both at home and outside home [23,55]. Indeed, BC patients are generally women, who may have to prepare meals for their families while coping with their taste alterations [10]. It has been reported that women with dysgeusia chose not to eat as much, impaired their eating time, and/or lost interest in preparing meals for themselves and/or their family [23]. Due to the early impact of dysgeusia on health, QoL, and social life of BC patients, devising strategies for prevention, early recognition, and treatment of taste impairment is of paramount importance [30].

### 3.4. The Strategies for the Management of Dysgeusia

The prevention and treatment of CT-related dysgeusia in patients with BC are limited. Several strategies have been proposed for preventing or ameliorating this symptom and its adverse and debilitating effects, but their efficacy is still controversial [57].

#### 3.4.1. Dietary and Educational Counseling

Dietary and educational counseling have been proven to alleviate the severity of dysgeusia in many cancers [5,58]. Available studies included mostly patients with head/neck or lung cancers, where aggressive therapies, such as surgery, chemotherapy, and radiotherapy led to severe oral complications, including dysgeusia [5,58]. A small single-center trial including gastrointestinal, breast, and lung cancer patients undergoing CT reported improvements in taste, from baseline to week 12, after intensified nutritional counseling with taste and smell training [8]. The general proposed nutritional recommendations for dysgeusia usually include: (1) increase fluid intake during meals to dissolve food components and translocate them more easily to the taste buds; (2) chewing food slowly to allow both the releasing of flavors and increased saliva production; (3) switch foods during meals to prevent taste bud adaptation; (4) flavoring foods with aroma enhancers, spices, and dressings; (5) reducing the consumption of bitter or metallic tasting foods (i.e., red meat, coffee); and (6) optimal oral hygiene and tongue brushing to improve taste acuity [57,58,59]. Overall, dietary interventions seem to provide modest benefits on treating the severity of dysgeusia of cancer patients [5,60]. Few studies on the effects of dietary counseling including only BC patients are at present available. A small qualitative study showed that, in BC patients treated with docetaxel or paclitaxel, dysgeusia affected both eating and food behaviors, pushing women to change their habits to cope and manage the taste alterations [23]. However, spontaneous strategies may not always be wholesome, leading, for example, to an increased use of salty foods, energy-dense condiments, sweets, or sugary drinks [23]. On the other hand, a small 9-weeks randomized controlled trial showed that self-monitoring (i.e., the observation and recording of the recognition, moods, and behaviors toward taste changes together with set goals for coping in collaboration with the researcher) determined a significant lower level of recognition of unpleasant taste in BC patients experiencing CT-induced taste alterations with respect to controls (i.e., education about taste alterations and oral care, and conventional nursing support for taste change management) [10]. Finally, patients with cancer frequently suffer from anxiety, depression, and sleep disturbances related to their diagnosis and the side effects of chemotherapy. These symptoms seem to be accentuated in the presence of treatment-related taste alterations [61]. Patients with these disturbances may have more difficulties in self-care, particularly in those behaviors which may enhance taste perception [62]. An educational intervention by audiotapes providing information about the nutritional management of side effects and exercise and relaxation techniques was proven to be effective in reducing anxiety, ameliorating self-care behaviors, and reducing dysgeusia in a small non-randomized trial of BC patients [62]. Therefore, informing patients about the possible adverse events from chemotherapy and providing them with advice regarding their management is important, but a great deal of attention should be paid to the psychological and emotional reactions of BC patients by giving the adequate support to obtain the maximum adherence to the lifestyle recommendations provided.

#### 3.4.2. Zinc Supplementation

Due to the relevant role of zinc in taste perception, its supplementation has been hypothesized to attenuate the dysgeusia of BC patients. However, its clinical efficacy is highly controversial in other types of cancer [63,64,65,66]. A double-blinded, placebo-controlled randomized clinical trial on 58 cancer patients (36% patients with BC) with dysgeusia or dysosmia did not find an improvement of taste or smell with the addition of zinc over 3 months [38]. However, in a prospective interventional study on 28 women undergoing CT for gynecological malignancies reporting dysgeusia after the first-line CT, the supplementation of zinc before CT promptly increased serum zinc level and prevented taste alterations, regardless of the drug administered [67]. Most studies had small sample size or methodological issues, employed different zinc dosages, and evaluated dysgeusia by self-reported assessments. Thus, evidence about the efficacy of zinc supplementation is still lacking and its benefit may be roughly considered at present as modest, even if subtle favorable effects not discerned by the available studies cannot be ruled out.

#### 3.4.3. Amifostine

Amifostine (AMF) is a thiol compound with cytoprotective activity used in radiotherapy for protection against xerostomia [68]. There is also strong evidence that amifostine reduces radiation-induced mucositis and pneumonitis [69]. Following rapid hydrolysis by the alkaline phosphatases, the active metabolite of amifostine (WR-1065) acts as a potent intra-cellular free radical scavenger, reducing DNA damage [70]. The parenteral treatment with AMF protects cells from normal tissues against DNA damage from both ionizing radiation and chemotherapeutic agents, while it does not protect cancer cells [71]. Differences in tumor tissue biology prevent either intracellular accumulation or cytoprotective function of AMF in cancer cells [71]. In mice, pretreatment with AMF reduced the amount of CYP-induced TNF-α expression in taste buds, suggesting that this drug is capable of protecting normal cells of the taste system from adverse effects of CYP [32]. In addition, in mice, pretreatment with AMF appeared to prevent CYP-induced loss of cells within taste buds and protect the cells involved in taste cell renewal after both single or fractionated doses of CYP [46]. These findings may have potential clinical implications for patients on CT; however, the effects of this drug on the prevention of human taste alterations after CT were minimal (for head and neck cancer) if not nil (unresectable non-small-cell lung cancer) [72,73] and no data on BC patients are available.

#### 3.4.4. Selenium

Selenium is an essential cofactor of the antioxidant enzyme glutathione peroxidase [74]. The supplementation with sodium selenite resulted in a milder, not significant, loss of taste when compared to no supplementation in a small RCT in patients with head and neck cancer undergoing radiotherapy [75]. No study has explored this potential benefit of selenium on CT-induced dysgeusia in BC.

#### 3.4.5. Lactoferrin

Lactoferrin, a natural iron-binding protein with high affinity for ferric (Fe^3+^) ions and immunomodulatory properties, is a key component of saliva because it maintains oral hygiene and acts as the first line immune defense in the oral mucosa [76]. In healthy volunteers, the administration of a lactoferrin solution completely removed the metallic flavor induced by the ingestion of ferrous containing water [77]. The authors hypothesized that lactoferrin, acting as a metal chelator, may reduce or eliminate the metallic sensation frequently perceived by cancer patients through binding iron ions and reducing iron-induced oxidative stress and lipid oxidation [77]. A small trial evaluated the efficacy of lactoferrin supplementation (250 mg, three times/day for 30 days) as a treatment for self-reported dysgeusia in 12 cancer patients (four with BC), who had developed self-reported taste and smell alterations after receiving CT compared with 12 healthy controls [78], showing a significant reduction in the scores for dysgeusia (2.0 points reduction of a score 0–10 based on nine questions on taste changes, *p* = 0.02) with respect to baseline at 30-day post-supplementation. The authors reported an increased expression of salivary immune proteins at proteomic analysis, which are associated with taste bud growth, neutral signal transduction, and taste threshold recovery [78]. Lactoferrin supplementation (750 mg daily for 30 days) was successfully reported to improve taste scores by 1.9 points (similar score 0–10, based on nine questions on taste changes, *p* = 0.001) among 26 cancer patients (five patients with BC) receiving CT in a single arm pilot trial [36].

#### 3.4.6. Cannabinoids

Cannabinoids are terpenophenolic compounds derived from the plant Cannabis sativa L. that interact directly with cannabinoid receptors, ion channels, and nuclear receptors comprising the endogenous endocannabinoid system, with reported anti-cancer effects [79,80,81]. Cannabinoids have been used to manage cancer-therapy-related symptoms such as nausea, vomiting, anorexia, anxiety, pain, and depression [82,83]. A phase II, randomized, double-blind, placebo-controlled pilot study explored the effect of delta-9-tetrahydrocannabinol (THC) in 21 patients with advanced cancer (one patient with BC) on dysgeusia and reported significant improvements in self-reported taste perception, appetite, and QoL [84]. Further larger studies are needed to clarify the potential use of cannabinoids in treating dysgeusia of BC patients.

#### 3.4.7. Dexamethasone

Dexamethasone is a long-acting corticosteroid recommended for chemotherapy-induced nausea and vomiting prevention [85]. A retrospective study of 131 BC patients receiving anthracycline-containing chemotherapy evaluated the impact of systemic administration of low-dose (*n* = 43) or high-dose (*n* = 88) dexamethasone for antiemetic purposes on oral mucositis and dysgeusia [85]. The incidence of dysgeusia was similar between the high-dose (9.9 mg infusion on day 1 and 8 mg orally on days 2–4) and low-dose (6.6 mg infusion on day 1 and 4 mg orally on days 2–4) groups [85]. No further studies have explored the effect of dexamethasone on CT-induced dysgeusia.

#### 3.4.8. Granulocyte Colony-Stimulating Factor

A phase III, open-label, randomized multicenter study on 1047 women with high-risk, node-negative BC, reported that the addition of a primary prophylactic granulocyte colony-stimulating factor (G-CSF) to postsurgical chemotherapy with docetaxel, doxorubicin, and cyclophosphamide (TAC) significantly reduced the incidence of dysgeusia (3.1 versus 7.9%, *p* = 0.0335) compared to TAC without G-CSF [86].

### 3.5. Additional Non Pharmacologic Strategies for Dysgeusia Management

#### 3.5.1. Photobiomodulation

The photobiomodulation therapy is the use of red or near-infrared light which exerts anti-inflammatory and analgesic effects and promotes wound healing, tissue repair, and neural function improvements [87]. PBMT has been used in cancer care (i.e., BC, head, and neck cancer) to prevent or manage a wide range of treatment-related toxicities, including oral mucositis, lymphedema, neuropathy, and radiodermatitis, but also dysphagia and dysgeusia [88]. Very little data are available on the use of PBMT for the management of dysgeusia. A phase II, randomized, triple-blind, placebo-controlled trial assessed the effectiveness of PBMT in addition to CT with doxorubicin-cyclophosphamide in 112 BC patients [3]. The patients randomly received PBMT (*n* = 56) or placebo (simulated PBMT; *n* = 56). PBMT-treated patients showed significant less objective (by taste scores) and subjective (by questionnaires) taste loss (*p* < 0.05), as well as significantly lower incidence of cachexia, anorexia, diarrhea, oral mucositis, and vomiting, as well as a higher QoL [3]. Anti-inflammatory and antioxidant benefits of PBMT toward dysgeusia was hypothesized [3]. A narrative review of the literature on the effects of PBMT as a supportive care to manage side effects of therapies in BC patients reported a beneficial effect on oral mucositis; that is, the inflammation of the oral mucosa caused by chemo and/or radiotherapy occurring in 20–40% of the patients undergoing conventional CT [89]. The safety and cost-effectiveness of PMBT on other-than dysgeusia side effects of CT was controversial [90], and the potential utility of PBMT in the management of dysgeusia in cancer patients remains at present uncertain [91].

#### 3.5.2. Hypoglossal Acupuncture

Acupuncture has been practiced in China for more than 3000 years as a traditional medicine and consists of stimulation of specific acupoints, considered to be lines of energy (meridians), along the skin of the body with fine needles. It is used in the treatment of various physical and mental diseases including dysgeusia [92]. A randomized placebo-controlled trial on 37 adults with idiopathic dysgeusia, treated with either acupuncture in selected body and ear points or sham acupuncture, reported a significant improvement in gustatory function in the intervention group, which remained stable over a period of ten weeks after completion of the treatment [92]. An ongoing randomized controlled trial aimed to compare a single acupuncture treatment with two active comparators (sham acupuncture and dietary recommendations) on 75 BC patients undergoing platinum- or taxane-based chemotherapy and reporting dysgeusia [33]. At present, the results are not yet available.

## 4. Limitations

The heterogeneity in the methods employed to assess dysgeusia, the small sample sizes of most studies, together with the great variations in the study designs, the chemotherapeutic regimen used, the timing of taste assessment during treatment, the comparator groups (cancer patients undergoing different antineoplastic schemes, patients with different types of cancer or healthy controls), and the reported analyses (within-group before/after treatment or between-group assessments) made it difficult to obtain definitive results and make recommendations for the clinical practice.

## 5. Conclusions

Dysgeusia is a frequent and disabling complication of CT in patients with BC [7,93]. Its real prevalence is difficult to define due to the complexity of its etiopathogenesis, the lack of a standardized method for its definition, and the heterogeneity of the available studies on this topic. However, dysgeusia is a condition that has a significant impact on the nutritional status of the patients and compromises their health and quality of life.

Despite this, healthcare professionals often overlook this disorder, either considering it a minor and transient problem not worthy of treatment or even not considering it at all. Unfortunately, the possibilities of treatment are currently scarce and often disappointing and at present the best approach remains dietary and educational counselling. Increased efforts for patient education and sensitization of clinicians toward this problem are therefore mandatory. There is an urgent need of clinical trials for specific interventions to better manage dysgeusia and avoid the related heavy consequences on health and quality of life.

## Figures and Tables

**Table 1:** Characteristics of the studies assessing the occurrence of CT-related dysgeusia.

Author, Year, Country [Ref]	Study Design	Study Population	Number of pts	Intervention (CT Scheme)	Methods to Detect Dysgeusia	Percentages of Dysgeusia
Bayo 2016, Spain [11]	cross-sectional study	women > 18 yo with BC	92	neoadjuvant or adjuvant TEC	questionnaires	prevalence of dysgeusia reported by oncologists: 4.5% vs. prevalence of dysgeusia reported by patients: 87%
Bernhardson 2008, Sweden [15]	multicenter, cross-sectional, observational study	patients > 18 yo with breast, gastrointestinal, gynecological, or other cancer	518 pts, 189 (36%) BC pts	different schemes of CT with either single agent or combination CT	questionnaires	prevalence of self-reported dysgeusia: 67% of the total sample, 83% of BC patients
Denda 2020, Japan [6]	prospective cohort study	patients > 20 yo with BC	41	EC therapy for a total of 4 times every 3 weeks	filter paper disk method assessment (objective assessment) and questionnaires (subjective assessment)	incidence of dysgeusia: 56% (23 out of 41 pts) by self-reported questionnaire and 34% (14 of 41 pts) by the FPD method on day 4 post-CT.
de Vries 2018, Netherlands [16]	multicenter observational study	women with newly diagnosed, stage I–IIIB, operable BC	135	different schemes of CT with or without trastuzumab	questionnaires and self-reported taste and smell perception	prevalence of dysgeusia 68% after 1 month and 16% after 6 months from the last CT cycle
Montemurro 2016, Italy [12]	multicenter, prospective, single-arm study	women > 18 yo after surgery for BC	604	standard adjuvant CT (not specified)	NCI CTCAE v4.0 (by pts) and medical records (by physicians)	incidence of dysgeusia: after the first cycle of CT 50% (277 of 556 pts) reported by pts and 8% (46 of 556 pts) by physicians, and after the third cycle of CT: 58% (314 of 537 pts) reported by pts
von Grundherr 2019, Germany [8]	single-center phase II trial	patients > 18 yo with breast, gastrointestinal, or lung cancer	62 pts, 31 (50%) BC pts	different schemes of CT with either single agent taxane, platinum, anthracycline, or fluoropyrimidine (19% [*n* = 12]), or combination CT (81% [*n* = 50])	taste strips test plus taste questionnaires	incidence of measured dysgeusia 48% (30 of 62 pts showed ≤8 points by taste strips); median taste strips in BC pts 10.0 points. Incidence of self-reported dysgeusia 77% (48 of 62 pts)
Gadisa 2020, Ethiopia [18]	prospective cohort study	women > 18 yo with BC	146	neo/adjuvant or palliative chemotherapy with AC or ACT	NCI CTCAE v4.0	incidence of dysgeusia: 97.3% (142 of 146 pts) totally, 91.7% with AC and 97.3% with ACT
Jensen 2008, Denmark [25]	prospective, controlled, observational study	women < 35 yo with early-stage BC	77 (46 CT), 31 (no CT)	adjuvant CT with CMF or FEC	interview	incidence of dysgeusia 84% (38 of 46 pts) under adjuvant CT
Kaizu 2021, Japan [17]	multicenter cross-sectional study	patients > 20 yo with breast or pancreas cancer	100 pts, 79 (79%) BC pts	paclitaxel, docetaxel, or nab-paclitaxel as monotherapy or combination therapy	taste strips test and questionnaires	prevalence of dysgeusia: 59% (59 of 100 pts) totally and 81.4% (48 of 79 pts) in BC pts; specifically, prevalence 95% in docetaxel-treated pts (total sample)
Kozloff 2010, USA [26]	exploratory nonrandomized study	women > 18 yo with locally advanced or metastatic BC	22	sunitinib + paclitaxel	NCI CTCAE v3.0	incidence of dysgeusia 68% (15 of 22 pts)
Miles 2013, 25 countries [27]	randomized, double-blind, placebo-controlled, phase III trial	patients > 18 yo with HER2-positive locally recurrent, unresectable, or metastatic BC	808	trastuzumab, docetaxel + pertuzumab or placebo	NCI-CTCAE v3.0	incidence of dysgeusia: 15.6% in placebo arm (62 of 397 pts) and 18.4% in pertuzumab arm (75 of 407 pts)
Nuzzo 2008, Italy [21]	multicenter, phase III, randomized trial	early BC patients 65–79 yo with average to high risk of recurrence	103	adjuvant CMF or docetaxel	NCI-CTC v2.0	incidence of dysgeusia after 1 cycle of CT: 11.3% in CMF and 25% in docetaxel group
Pedersini 2022, Italy [20]	prospective single-center study	women 65–79 yo operated for BC, with average to high risk of recurrence	299	adjuvant CT with docetaxel versus CMF	NCI CTC v2.0	docetaxel induced more frequent/severe dysgeusia compared to CMF
Perrone 2015, Italy [22]	multicenter, randomized, phase III study	women 65–79 yo operated for BC with average to high risk of recurrence	299	adjuvant CT with docetaxel versus CMF	NCI CTC v2.0	docetaxel induced more frequent/severe dysgeusia compared to CMF
Ponticelli 2017, Italy [13]	cross sectional study	patients 18–80 yo with solid or haematologic cancer	289 pts, 59 (20.4%) BC pts	different schemes of CT	questionnaires	prevalence of dysgeusia: 64% (185 of 289 pts) totally and 69.5% (41 of 59 pts) in BC pts
Saini 2015, India [28]	observational prospective study	patients > 18 yo with breast or lung cancer	174 pts, 101 (58%) BC pts	multiple schemes: - FAC (41.85%) - paclitaxel (22.77%) - docetaxel (10.89%) - FEC (9.90%) - other regimens	patient interview	incidence of dysgeusia 38.5% in BC pts treated with single/combined regimen
Speck 2013, USA [23]	exploratory descriptive study	women 21–70 yo with BC who completed two CT cycles or within 6 months post-CT	25	neoadjuvant, adjuvant, or palliative (metastatic BC) docetaxel or paclitaxel	semi-structured interviews and patient-level data	incidence of dysgeusia 44% (11 of 25 pts: 8 of 10 docetaxel pts and 3 of 15 paclitaxel pts)
Steinbach 2009, Germany [29]	multicenter prospective cohort study	patients with breast or gynecologic cancer	87 pts, 69 (79%) BC pts	multiple schemes: - anthracycline and taxane containing CT (45%) - platinum-containing CT (27%) - FEC (23%) - CMF (5%)	taste strips	gustatory function significantly decreased during chemotherapy (more with taxane-based CT)

Abbreviations: AC: doxorubicin-cyclophosphamide; ACT: doxorubicin-cyclophosphamide-paclitaxel; BC: breast cancer; CiTAS: chemotherapy-induced taste alteration scale; CMF: cyclophosphamide, methotrexate, fluorouracil; CT: chemotherapy, CTCAE: Common Terminology Criteria for Adverse Event, CTC: common toxicity criteria; FAC: 5-fluorouracil, doxorubicin, cyclophosphamide; EC: epirubicin and cyclophosphamide; FEC: fluorouracil, epirubicin, and cyclophosphamide; FPD: filter paper disk; NCI: National Cancer Institute; pts: patients; PPG: primary prophylactic G-CSF; TAC: docetaxel, doxorubicin, cyclophosphamide; yo: years old.
